# Bilateral Focal Choroidal Excavation and Central Serous Chorioretinopathy Coexisting in a Male Patient

**DOI:** 10.1155/2024/5519361

**Published:** 2024-03-26

**Authors:** Jie Bai, Yanqing Wang, Nanjue Cao, Yan Liu, Xufei Chen, Ting He, Shan Wang

**Affiliations:** ^1^Department of Ophthalmology, The Fourth Affiliated Hospital, Zhejiang University School of Medicine, Yiwu 322000, Zhejiang, China; ^2^Department of Ophthalmology and Otorhinolaryngology, Yiwu Second People's Hospital, Yiwu 322000, Zhejiang, China; ^3^Department of Oral Pathology, School of Stomatology, Hainan Medical University, Haikou 571199, China

## Abstract

**Background:**

Here, we report a case of a male patient with bilateral focal choroidal excavation (FCE) and central serous chorioretinopathy (CSC). A 33-year-old man complained of mild blurring of vision in the right eye. Optical coherence tomography (OCT) revealed FCE in both eyes, with subretinal fluid in both eyes and serous pigment epithelial detachment in the right eye. Standard laser fluence (50 J/cm^2^) was used in the right eye, and a subthreshold micropulse laser (SML) was simultaneously used in the left eye. Follow-up visits were recommended. At his last visit (5 months after treatment), the visual acuity was 16/20 in the right eye and 20/20 in the left eye and OCT showed a completed resolution of SRF.

**Conclusion:**

FCE is defined as a localized depression of the choroid detected by OCT. It may be congenital or acquired secondarily. We present a case of uncommon focal choroidal excavation and central serous chorioretinopathy (CSC) coexisting in both eyes at a relatively young age in which visual acuity was improved and subretinal fluid (SRF) completely resolved with laser treatment. Timely treatment can promote SRF absorption and improve vision.

## 1. Introduction

Focal choroidal excavation (FCE) is a relatively uncommon concavity in choroids with unknown etiology. Patients tend to be asymptomatic and show minimal changes over time; however, a few patients report metamorphopsia or slightly decreased vision [1, 2]. Optical coherence tomography (OCT) is a reliable diagnostic technique [3, 4]. Here, we report the case of a patient with FCE who presented with coexisting central serous chorioretinopathy (CSC) in both eyes.

## 2. Case Report

**Figure 1:**
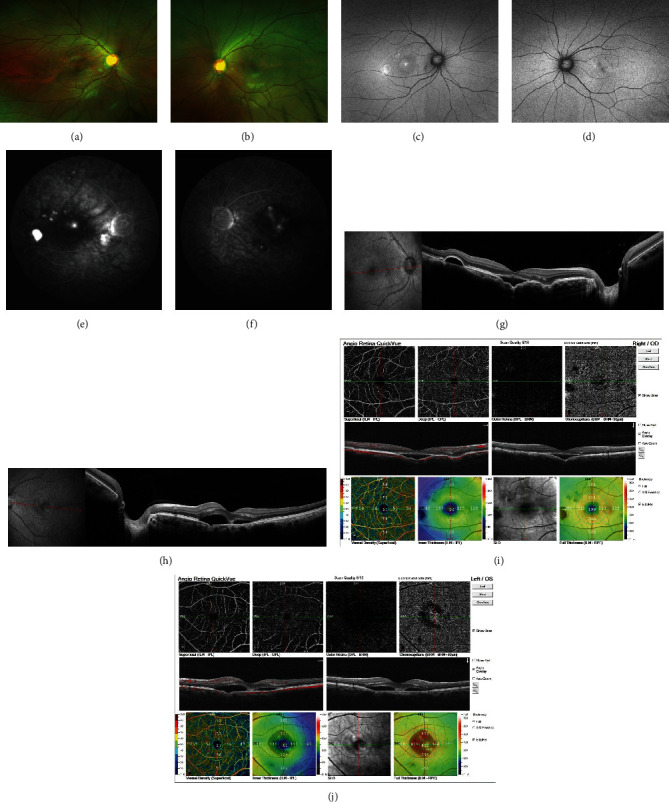
(a, b) At the first visit, color fundus photographs taken on the Optos platform showed mild perifovea pigmentary change. (c, d) AF showed markedly increased AF. (e, f) FFA appeared as multifocal lesions of hyperfluorescence in the late stage. (g) OCT revealed nonconforming FCE with retinal neuroepithelium and retinal pigment epithelium serous detachment in the right eye. (h) OCT showed nonconforming FCE with retinal neuroepithelium serous detachment in the left eye. (i, j) OCTA showed no evidence of neovessel in the right or left eye.

**Figure 2:**
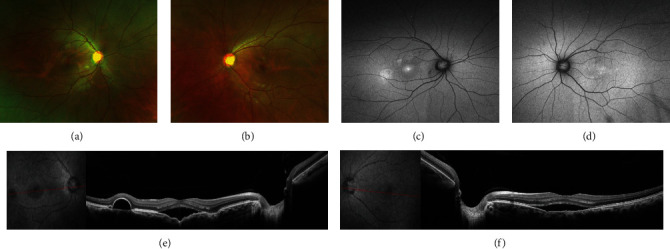
(a, b) Color fundus photographs taken 2 months after his first visit showed stable perifoveal pigmentary retinal changes in both eyes. (c, d) AF demonstrated increased hyperautofluorescence compared to before. (e) OCT revealed increased SRF in the right eye after 2 months of follow-up. (f) OCT showed that the serous fluid in the left eye was stable.

**Figure 3:**
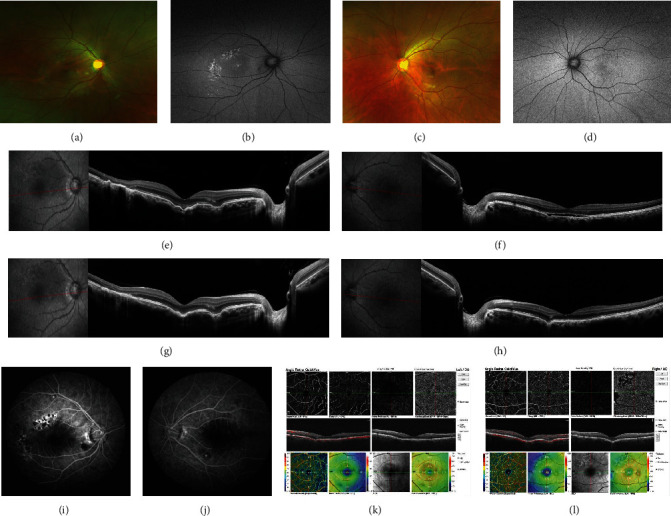
(a) Color fundus of the right eye showed perifoveal retinal pigmentary changes 1 month after his treatment. (b) AF demonstrated scattered dots with high fluorescence increases corresponding to the site of laser treatment. (c) Color fundus of the right eye showed no change. (d) AF of the right eye showed no change. (e, f) Follow-up OCT showed the serous fluid in both eyes resolved markedly. (g, h) Follow-up OCT at 4 months showed a completed resolution of SRF after treatment, and the FCE remained the same. (i, j) There was no active leakage in the FFA. (k, l) OCTA showed no evidence of neovessel in the right or left eye.

A 33-year-old man complained of decreased vision in the right eye over the previous 6 days. The patient had no history of steroid use; his refraction was −0.75 sphere in the right eye and −0.50 sphere in the left. The best-corrected visual acuity (BCVA) was 20/25 in the right eye and 20/20 in the left eye. The anterior segments of both eyes were unremarkable. Examination of both the fundi revealed mild perifoveal retinal pigmentary changes, with greater changes in the right eye (Figures 1(a) and 1(b)). Fundus autofluorescence (AF) revealed areas corresponding to retinal lesions (Figures 1(c) and 1(d)). FCE appears as fluorescence transmitted, and multifocal lesions showed hyperfluorescence in the late stage of fluorescein fundus angiography (FFA) (Figures 1(e) and 1(f)). Optical coherence tomography (OCT) revealed FCE with subretinal fluid in both eyes and serous pigment epithelial detachment in the right eye (Figures 1(g) and 1(h)). Optical coherence tomography angiography (OCTA) showed no evidence of flow signals in the right or left eye (Figures 1(i) and 1(j)). The patient was diagnosed with nonconforming FCE associated with CSC. He returned 2 months later for a follow-up visit, with a visual acuity of 20/40 in the right eye and 20/20 in the left eye. There seem no obvious changes in fundoscopy (Figures 2(a)–2(d)), OCT revealed increased SRF in the right eye compared with that in his first visit (Figure 2(e)), and the serous fluid in the left eye was stable (Figure 2(f)). Standard laser fluence (level: I spot; spot diameter: 50 *μ*m; power: 70 mW; exposure time: 0.1 s.) was used in the right eye, and a subthreshold micropulse laser (SML) was simultaneously used in the left eye. One month after treatment, the subretinal fluid had resolved, and visual acuity was 20/30 in the right eye and 20/20 in the left eye. At his last follow-up (5 months after laser treatment), his visual acuity was 16/20 in the right eye and 20/20 in the left eye, with no recurrence of the subretinal fluid. There was no active leakage in the FFA (Figure 3).

## 3. Discussion

FCE is defined as a localized depression of the choroid detected by OCT, its etiology is not fully understood and it is characterized by good visual acuity, and few people show decreased vision or metamorphopsia [5]. FCE belongs to the spectrum of diseases associated with the pachychoroid spectrum. Ellabban et al. believe that the choroidal thickness in patients with FCE complicated by central serous chorioretinopathy (CSC) is greater than in normal eyes [6].

Park and Oh conducted a study on the prevalence of FCE and found that among 1,697 patients (under 40 years old) visiting ophthalmology clinics, only 3 cases were FCE patients, but whether the low incidence reflects that FCE is related to acquired factors needs further investigation [7]. FCE usually affects one eye, but there have also been reports suggesting that some patients may have bilateral disease. In Zheng-Yu et al.'s study, out of 18 patients, 7 (38.89%) had bilateral involvement, and two patients had two excavations in one eye [8]. The incidence of FCE in patients with CSC ranges from 2.8% to 7.8% [9]. In addition to CSC, FCE may accompany other diseases, including choroidal neovascularization (CNV), bestrophinopathies, age-related macular degeneration (AMD), and polypoidal choroidal vasculopathy (PCV) [10].

Whether CSC occurs as a complication of excavation or leads to FCE has not been determined. FCE may represent either the sequelae of an unidentified chronic process or congenital abnormalities. Matsubara et al. postulated that atrophic RPE at the site of the FCE lesion and choroidal circulatory disruption may be related to FCE complicated by CSC [11]. Studies have found that in the excavated area of FCE, the choroidal capillary layer exhibits a dark flow signal void area on OCTA, surrounded by a high perfusion area. FFA suggests that leakage points can be seen at the edges of FCE, and strong fluorescence spots appear in the late phase, further indicating that choroidal circulation disorders and RPE layer atrophy are key factors in its pathogenesis.

Although most FCE cases remain stable for a long time, researchers still suggest that changes in choroid that occur in FCE may lead to the development of CNV [12]; therefore, lifelong monitoring is needed for patients with FCE. Promoting subretinal fluid absorption as soon as possible is a key goal in patients with CSCs. Fluid in CSC is due to leakage; as a result, the detachment between the retinal epithelium and RPE layers could be larger than the excavation [13]. In this report, after 2 months of observation, subretinal fluid increased, despite the progressively decreasing vision, the patient's right eye was treated with an FFA-guided focal laser to the point leaks, and the left eye was treated with a micropulse laser. Chen et al. used standard laser and half-dose PDT to treat patients with FCE and active CSC, which achieved complete resolution of SRF and vision improvement despite the persistence of FCE after treatment [14]. Our study showed similar results.

## Data Availability

The datasets used and/or analyzed during the present study are available from the corresponding authors on reasonable request.
